# Nano-biochar: recent progress, challenges, and opportunities for sustainable environmental remediation

**DOI:** 10.3389/fmicb.2023.1214870

**Published:** 2023-07-19

**Authors:** Geeta Bhandari, Saurabh Gangola, Archna Dhasmana, Vishal Rajput, Sanjay Gupta, Sumira Malik, Petr Slama

**Affiliations:** ^1^Department of Biosciences, Himalayan School of Biosciences, Swami Rama Himalayan University, Dehradun, India; ^2^School of Agriculture, Graphic Era Hill University, Bhimtal Campus, Uttarakhand, India; ^3^Amity Institute of Biotechnology, Amity University Jharkhand, Ranchi, Jharkhand, India; ^4^Guru Nanak College of Pharmaceutical Sciences, Dehradun, Uttarakhand, India; ^5^Laboratory of Animal Immunology and Biotechnology, Department of Animal Morphology, Physiology and Genetics, Faculty of AgriSciences, Mendel University in Brno, Brno, Czechia

**Keywords:** Nano-biochar, biochar, nanotechnology, environmental pollution, remediation

## Abstract

Biochar is a carbonaceous by-product of lignocellulosic biomass developed by various thermochemical processes. Biochar can be transformed into “nano-biochar” by size reduction to nano-meters level. Nano-biochar presents remarkable physico-chemical behavior in comparison to macro-biochar including; higher stability, unique nanostructure, higher catalytic ability, larger specific surface area, higher porosity, improved surface functionality, and surface active sites. Nano-biochar efficiently regulates the transport and absorption of vital micro-and macro-nutrients, in addition to toxic contaminants (heavy metals, pesticides, antibiotics). However an extensive understanding of the recent nano-biochar studies is essential for large scale implementations, including development, physico-chemical properties and targeted use. Nano-biochar toxicity on different organisms and its in-direct effect on humans is an important issue of concern and needs to be extensively evaluated for large scale applications. This review provides a detailed insight on nanobiochar research for (1) development methodologies, (2) compositions and properties, (3) characterization methods, (4) potentiality as emerging sorbent, photocatalyst, enzyme carrier for environmental application, and (5) environmental concerns.

## Graphical abstract: Fabrication, characterization, and applications of nano-biochar.

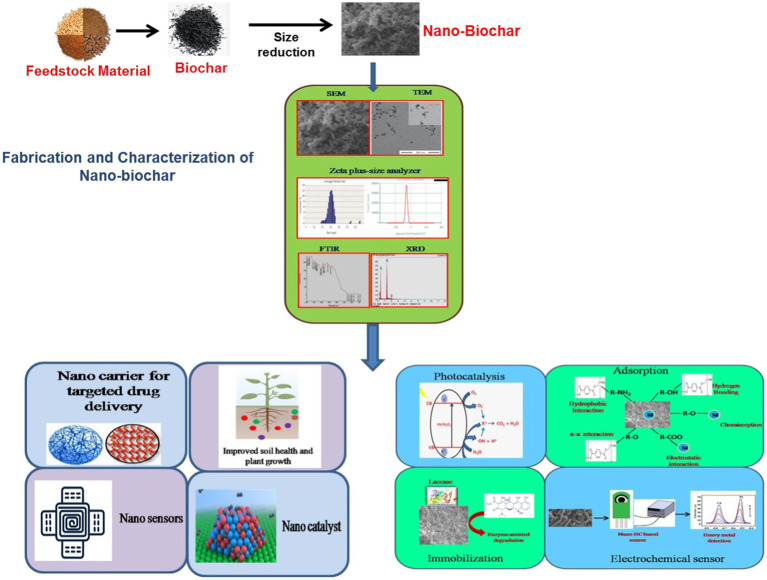

## 1. Introduction

Extensive industrialization, urbanization, and modern agricultural methods have resulted in accumulation of innumerous toxic compounds (pesticides, pharmaceutical and personal care products, antibiotics, hormones, organic compounds, nano-compounds, endocrine disruptors, steroids, surfactants and their metabolites, industrial additives, and heavy metals) in the different environmental matrices (Bhatt et al., 2022; Gangola et al., 2022). Anthropogenic activities such as healthcare, industries, power plants, oil refineries, mining, improper waste treatment, agriculture and household activities can lead to build-up of pollutants ranging from 1 μg/kg to 10 mg/kg in the different environments (Zhou et al., 2021). Furthermore, aquatic and soil sediments also function as potent sink for innumerous hydrophobic compounds (polychlorinated biphenyl, poly-and perfluoroalkyl compounds, and organochloride insecticides) (Bhatt et al., 2022). The immoderate enhancement in the amount of such contaminants in the environment has alarmed the scientific and regulatory bodies across the globe due to acute and chronic human health toxicities. With time, various physico-chemical and biological processes such as adsorption, advanced oxidation methods, sonocatalysis, nano-filtration/reverse osmosis and bioremediation have been developed for efficient treatment of contaminated environments (Amusat et al., 2021). However, majority of these advanced methods are energy and cost extensive and release more toxic secondary by-products in the environment. The sustainable, eco-friendly nature, easy operation, and low cost of bioremediation in comparison with traditional and advanced physico-chemical methods have resulted in the establishment of bioremediation technologies recently (Bhatt et al., 2020; Suresh et al., 2022). Nonetheless, limitations such as dynamic microbial habitat fluctuation, reproducibility, cross contamination with other contaminants, and interfacial physical and biogeochemical methods in the soil-aquatic shift may render biodegradation slow and inefficient (Mukherjee et al., 2022).

Several authors have recently concentrated on the utilization of nano-compounds for the development of better remediation methods (Mahmoud et al., 2022; Rajput et al., 2022). Due to distinct physical characters of nano-materials such as excellent surface-to-volume proportion, higher reactivity, ability modify surface chemistry, smaller intra-particle diffusion distance, higher contaminant removal efficiency, stable nature and reusable and recyclable capacity, nanobiotechnology has recently received great attention for environmental applications recently (Xia et al., 2022). Biochar (BC) is a carbon containing solid compound fabricated by pyrolytic degradation of biomass (agricultural, animal and solid waste) in the absolute vacuum conditions (Bolan et al., 2022). It is generally produced using different thermochemical methods; fast and slow pyrolysis, flash and hydrothermal carbonization, gasification and torrefaction (Bolan et al., 2022; Mukherjee et al., 2022). Biochar has shown a substantial ability to remediate pollutants since it is cheap accessibility of feedstock, economical, and desirable physicochemical surface properties (Xiao et al., 2021). Among these physicochemical properties, the biodegradable nature plays a crucial role especially in agricultural activities (Širić et al., 2022). The synthesis and applications of biochar have however also faced few hurdles due to low catalysis, inadequate pore size and surface area, deficiency of simple and chemical-free functionalization processes (Li et al., 2019a).

Recently, studies on the production of nano-biochar (nano-BC) for environmental and agriculture applications has been documented (Nath et al., 2019; Li et al., 2019b; Rajput et al., 2022). Carbonization results in fabrication of micro-sized BC with size 1 μm-1 nm referred to as “dissolved” or “nano-BC.” The elemental composition, aromatic/polar nature, cation exchange capacity, crystalline form, graphitic nature, pH, specific surface area, pore size, stability, temperature-dependent dispersibility and zeta potential of nano-BC vary in comparison with bulk-BC (Ramanayaka et al., 2020a). Colloidal and nano-BC possess features such as surface hydrophobicity, nano-scale size, significantly high specific surface area, micro-porous structure, diverse surface functionality (hydroxyl, carboxy, lactonyl) and thus significantly enhance the adsorption and immobilization capability of nano-BC for different pollutants, including heavy metals, pesticides, PCBs, PAHs, and others (Nath et al., 2019; Mahmoud et al., 2022). Nano-BC assisted adsorption for the removal of toxicants from water bodies have been developed recently, which also enable for both “C” sequestration in addition to remediation (Xia et al., 2022).

Furthermore, due to high porosity, surface functionality and larger surface-to-volume ratio, nano-BC functions as an excellent immobilization material for enzymes and can thus function as a nanocatalyst in bioremediation (Naghdi et al., 2018). The chemical and physical properties of nano-BC dictate its ability to remove various pollutants, which are dependent on feedstock material, production method, pyrolysis temperature, and other pre-or post-treatment methods (Xia et al., 2022). Thus, nano-BC, with its unique features and applications, opens up new avenues for a long-term, cost-effective, and sustainable solution to environmental pollution. Therefore, the present review provides updated information on the methodologies for fabrication and characterization of nano-BC and its application for managing hazardous contaminants in the environment. Furthermore, for future research, an extensive appraisal of the potentiality of nano-BC-assisted contaminant removal is presented.

## 2. Production of nanobiochar

**Figure 1:**
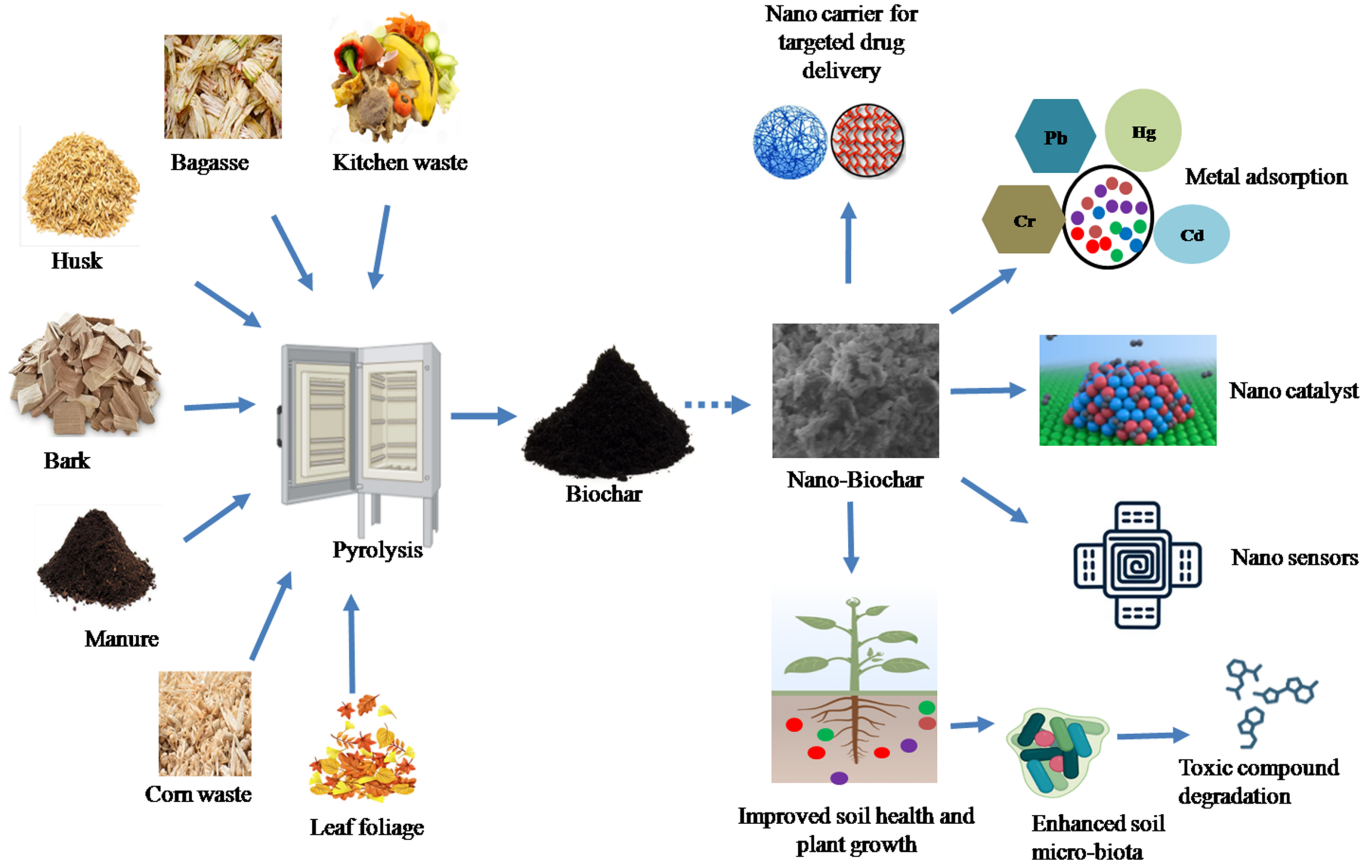
Different feedstock material for fabrication of nano-BC and its wide applications.

Nano-BC is a novel nano-sized carbonaceous material generally manufactured using green and energy-saving nanotechnology methods. Nano-BC differs from macrochar by of possessing higher specific surface area, higher porosity, lower hydrodynamic radius, stronger negative zeta potential, better oxygen-consisting surface functional groups, and lower carbon defects (Qin et al., 2018; Ramanayaka et al., 2020a). The most widely employed feedstocks for fabrication of nano-BC include animal wastes, municipal wastes, lignocellulosic agricultural wastes (grass, palm, peanut shell, rice husk and straw, sugar cane bagasse, bamboo, and soy bean stover), woody forest residues and sewage sludge. Initially the biomass is transformed into bulk-BC followed by size reduction through various fractionation approaches to produce nano-BC (Figure 1).

### 2.1. Preparation of bulk biochar

Biochar is fabricated from lignocellulosic biomass using thermo-chemical approaches such as pyrolysis (slow and fast), torrefaction, carbonization (hydrothermal or flash), and gasification (Amusat et al., 2021). The feedstock material is thermochemically or pyrolytically decomposed at 350–700°C in vacuum (*<*1% O_2_) for the generation of BC. Slow pyrolysis is an eco-friendly process commonly employed for BC fabrication and results in high yield of bio-oil and 35% yield of dry mass (Tomczyk et al., 2020). Zhang et al. (2017) used slow pyrolytically produced BC for soil remediation and sorption of different pollutants from wastewater. For biofuel production, fast pyrolysis is favored over other processes; gasification is primarily employed for the synthesis of syngas, which subsequently produces heat and energy. Additionally, lignocellulosic material caused higher BC yield than municipal solid waste (Ashiq et al., 2019). To expedite the nano-BC production the employment of BC produced by traditional thermochemical methods is advised, while optimizing the quality of biomass materials through transforming them to nano-particles. The traditional methods produce BC with different yields and elemental constitution (C, H, O). Biomass normally becomes more carbonized as treatment intensity increases, which corresponds to an elevation in C composition but a reduction in O and H composition. Additionally, BC is modified physically and chemically for a variety of purposes to enhance its functionality. Steam coating, chemical oxidation, acidic/basic treatment, CO_2_ activation, saturation with native and artificial nano-materials is used for chemically modifying BC (Song et al., 2022).

### 2.2. Conversion of bulk biochar to nanobiochar

Nano-BC inherently forms while synthesizing macro-BC, however its output is limited (<2.0% from peanut shell-derived BC) (Liu et al., 2018). It is thus necessary to undergo a size reduction process in order to enhance the amount of nano-BC (Table 1). The production of nanomaterials can be performed by top-down or bottom-up processes. In the top-down process, the size of the macro BC is minimized to nanoscale; whereas in the bottom-up method, the nanomaterial is amassed from the atomic level. Top–down methods such as grinding, cutting, centrifugation and etching are used for the fabrication of nano-BC in an economical manner. Lonappan et al. (2016), Dong et al. (2018), Lian et al. (2020), and Ramanayaka et al. (2020a) have employed grinders for reducing the size of macro-BC to nanoscale. The bottom up method includes disintegration by ball milling or sonication and carbonization. Ball milling enables fabrication of nano-BC with improved properties without destroying its crystal structure (Amusat et al., 2021). Ball-milling has received great attention because of its low cost and energy demand during manufacturing, eco-friendly nature and wide range of application. The ball milling method disintegrates bulk-BC into nanoscale by the colliding it between metallic balls. The desired particle size may be attained by regulating the aggregation and modifying the ball sample ratio and milling time. There are two ways to ball mill BC at the nanoscale: wet and dry techniques. The wet approach is more preferred due to synthesis of nano-BC with superior dispersivity, higher surface functionality, eco-friendly and less labor intensive approach (Yuan et al., 2020). Ball milling method effectively tailors nano-BC characteristics by enhancing surface area, decreasing material size, improving surface oxygen functionality, and increasing sorption and photocatalytic efficacy (Lyu et al., 2017, 2018; Naghdi et al., 2017; Wang et al., 2018). Nano-BC with particle size smaller than 100 nm was fabricated within 30 h using planetary ball milling method (Richard et al., 2016). Naghdi et al. (2019) suggested a pre-treatment of BC at 80°C for 24 h before conversion to nano-BC using planetary ball milling within 100 min. The pre-conditioning subdued the agglomeration of nano-particles and reduced the size of BC from 212 to 60 nm. Double-disc milling is also an alternative method for nano-BC fabrication; however it demands high operational costs. Among the different ball mill methods, vibrating disc milling produces greater quantities of nano-BC with consistent size and shape due to attrition and shear stress (Karinkanta et al., 2018). Several studies have reported fabrication of nano-BC in a controlled environment using process parameters such as milling period of 120–1,200 min, number of balls from 25 to 800, ball weight from 0.5 to 100 g, and ball size from 3/4 to 15 mm. Ma et al. (2022) optimized the process parameters for synthesis of ball-milled nano-BC by regulating grinding time, rotating speed, and ball-to-powder mass ratio. The BC mixtures must be subsequently dispersed in different solvents post milling to improve particle distribution before separation (Song et al., 2022). The pre-treatment at 80°C however enables the reduction in size aggregation, but is a high-cost method thus restricting its scale-up. Iron oxides can be also added into BC for suppressing the agglomeration of particles and enhancing their distribution (Li et al., 2020a). Ball milling is a high atom economy method and generates nano-scale biodegradable products using renewable sources by limiting the usage of hazardous chemical-assisted procedures.

**Table 1:** Different approaches for synthesis of nano-biochar and its wide applications.

Feedstock	Methodology	Nano-BC characteristics	Application	Performance	References
Pine wood	Planetary ball miller	Nano-BC (60 ± 20 nm)	Elimination of carbamazepine	95% removal of carbamazepine	Naghdi et al. (2019)
Rice husk	Ball milled nano-BC treated by one-pot pyrolytic method	Iron oxide permeated mesoporous nano-BC	Adsorbent for As	>90% adsorption of As	Nath et al. (2019)
Microcrystalline cellulose	*In situ* precipitation and carbonization	ZnO modified nano-BC	Photocatalyst for elimination of Phenol	99.8% removal of phenol within 90 min	Zhang et al. (2020)
Wheat straw	Ball-milled at 700°C	Magnetic nano-BC	Adsorbent for f tetracycline and Hg	Adsorption rate of 268.3 mg g^−1^(tetracycline) and 127.4 mg g^−1^ (Hg)	Li et al. (2020a)
Soybean straw and cattle manure	Digestion of the bulk-BC in high pressure and acidic conditions in a hydrothermal reactor	BC nanodots, (4–5 nm)	–	–	Guo et al. (2020)
Wood BC (a by-product of *Gliricidia sepium* gasification)	pre-treated BC (at – 80°C for 3 days) in ethanol media was disc milled	Graphitic nano-BC (surface area of 28 m^2^/g and high surface functionality)	Elimination of oxytetracycline, glyphosate, Cr (VI) and cadmium (Cd (II))	High partition coefficient in comparison to other adsorbents for the elimination of contaminants	Ramanayaka et al. (2020a)
Wood BC (a by-product of *Gliricidia sepium* gasification)	pre-treated BC (at – 80°C for 3 days) in ethanol media was disc milled	Graphitic nano-BC (surface area of 28 m^2^/g and high surface functionality)	Adsorbent for Oxytetracycline	A two-step sorption with sorption rate of 16.9 and 113.2 mg g^−1^	Ramanayaka et al. (2020b)
Oil palm	Pyrolysis-carbonization of FeCl_3_ pre-treated biomass at 500°C and sulfonation	Sulphonated magnetic nano-BC in amorphous phase with crystallite Fe_3_O_4_	Acid catalyst	High catalytic activity in comparison to commercial catalysts for esterification	Jenie et al. (2020)
Waste lignin	High temperature carbonization	Nano-BC (vesicular, specific surface area of 83.41 m^2^/g)	Alternative of carbon black	Renewable filler of styrene-butadiene rubber	Jiang et al. (2020)
*Cynodon dactylon* (L.) pers. residues	Hydrothermal and co-precipitation method	Amino-substituted silica-coated nano-BC (0 nm size, spherical shape and superparamagnetic nature)	Adsorbent for Cu^2+^ and Pb^2+^	Adsorption rate of 220.4 mg g^−1^ (Cu^2+^) and 180.5 mg g^−1^ (Pb^2+^)	Vishnu et al. (2021)
Wheat	Impregnation	Wheat nano-BC	Nanofertilizer	Slow release of nitrate, phosphate, potassium and sodium	Khan et al. (2021)
*Cynara scolymus* L. leaves	Pyrolysis at 350°C for 1 h	Ecofriendly nano-BC	Nanoadsorbent for Cd and Sm by microwave sorption	Sorption rates of 1,150 μmol g^−1^ (Cd^2+^) and 650 μmol g^−1^ (Sm^3+^)	Mahmoud et al. (2021)
Orange peel	Hydrothermal carbonization	Graphene based nano-BC (10–100 nm, high surface functionality, fluorescent and high water-dispersion)	Biocompatible nanocarrier	Capable of targeted cancer therapy	Iannazzo et al. (2022)
Orange peel waste derived hydrochar	Hydrothermal carbonization	Nano-BC	Electrochemical sensor	Detection of sulfites and nitrites in wastewater	Ferlazzo et al. (2023)
Goat manure	Ball miller	Nano-BC (0 nm and high surface functionality)	Nanofertilizer	Improved soil microflora, soil health, and wheat production	Rashid et al. (2023)
*Cynara scolymus* leaves	Gentle milling and surface modification with Amberlite cation exchanger (ACE) IR-120	Immobilized ACE nano-BC (18.74–23.70 nm)	Nanobiosorbent	Removal rates of 91.74–98.19% (Pb^2+^) and 96.27–99.14% (methylene blue)	Mahmoud et al. (2023)

Among physical methods, sonication is an efficient method for production of nano-BC by employing high-energy ultrasonic radiations to disintegrate BC in suspension. The microporous region in BC increases due to shock waves resulting in opening of clogged pores and exfoliating the carbon structure. The small exfoliated particles then adhere to the surface or embed in the pores of BC resulting in nano-BC production (Liu et al., 2018). The uniformity in nano-BC surface and the development of porosity without obstruction are two prime benefits of sonication (Yang Y. et al., 2020). Few investigations also reported the generation of nano-BC from waste lignin carbonization as a post or pre-treatment with milling for enhancing the surface features and size of nano-BC with subsequent removal of impregnating salts (Jiang et al., 2020; Makshut et al., 2020). Guo et al. (2020) employed hydrothermal reaction for fabricating nano-BC from agricultural waste biomass. Soybean straw and animal wastes were employed as feedstock and digested with acids in a high-pressure hydrothermal reactor. Furthermore, multiple rounds of centrifugation are also employed for separating highly dispersed nano-BC particles (Ullmann et al., 2017). Different feedstock material and pyrolytic conditions, centrifugation period (2–30 min) and rotational speed (3,500–1,000 rpm) were used to prepare nano-BC (Anupama and Khare, 2021).

### 2.3. Functionalization of nano biochar

The intrinsic characters of nano-BC can be readily modified thus providing a platform for the easy modification for wide applicability in different sectors. Surface fictionalization using amination, sulfonation and oxidation improves the performance of BC-based nanomaterials (Nath et al., 2019). It has been observed that employment of different combination of pure and acid mixtures (H_2_SO_4_, HNO_3_, and HCl) for surface functionalization increased carboxylic group formation with concurrent laccase adsorption (Naghdi et al., 2017). Similarly, Fe_3_O_4_ engineered nano-BC has a greater surface area and adsorption site owing to mesoporous structure (Nath et al., 2019). Cellulosic nanocrystal derived nano-BC was modified with ZnO and it displayed greater active sites and functioned as potent photo-catalysts for phenol removal (Zhang et al., 2021). Nano-BC obtained from artichoke leaves was base-modified with NaOH and employed for removal of metformin hydrochloride. The results revealed the existence of COOH, OH, and C=C groups and higher elimination rates of metformin by modified nano-BC in comparison with pristine nano-BC (Mahmoud et al., 2020). Ethylenediamine functionalized nano-BC was employed as an effective nano-sorbent for removing prednisolone and Cr(VI) (Mahmoud et al., 2022). The significance of magnetic nano-BC for treating tetracycline and Hg(II) polluted wastewater was assessed by Li et al. (2019b). The modified nano-BC exhibited high removal rates (>99%) for both tetracycline and Hg(II) (Li et al., 2019b). The employment of pristine BC imposes some limitations on the adsorption efficacy for various contaminants due to low surface functionality and pore size. The surface modification of BC by different approaches improves the surface area, furnishes additional surface functional groups and adsorption sites. Thus, functionalized BC is a promising potential substitute for treating wide range of contaminants.

## 3. Inherent properties of nano-BC and their characterization

**Figure 2:**
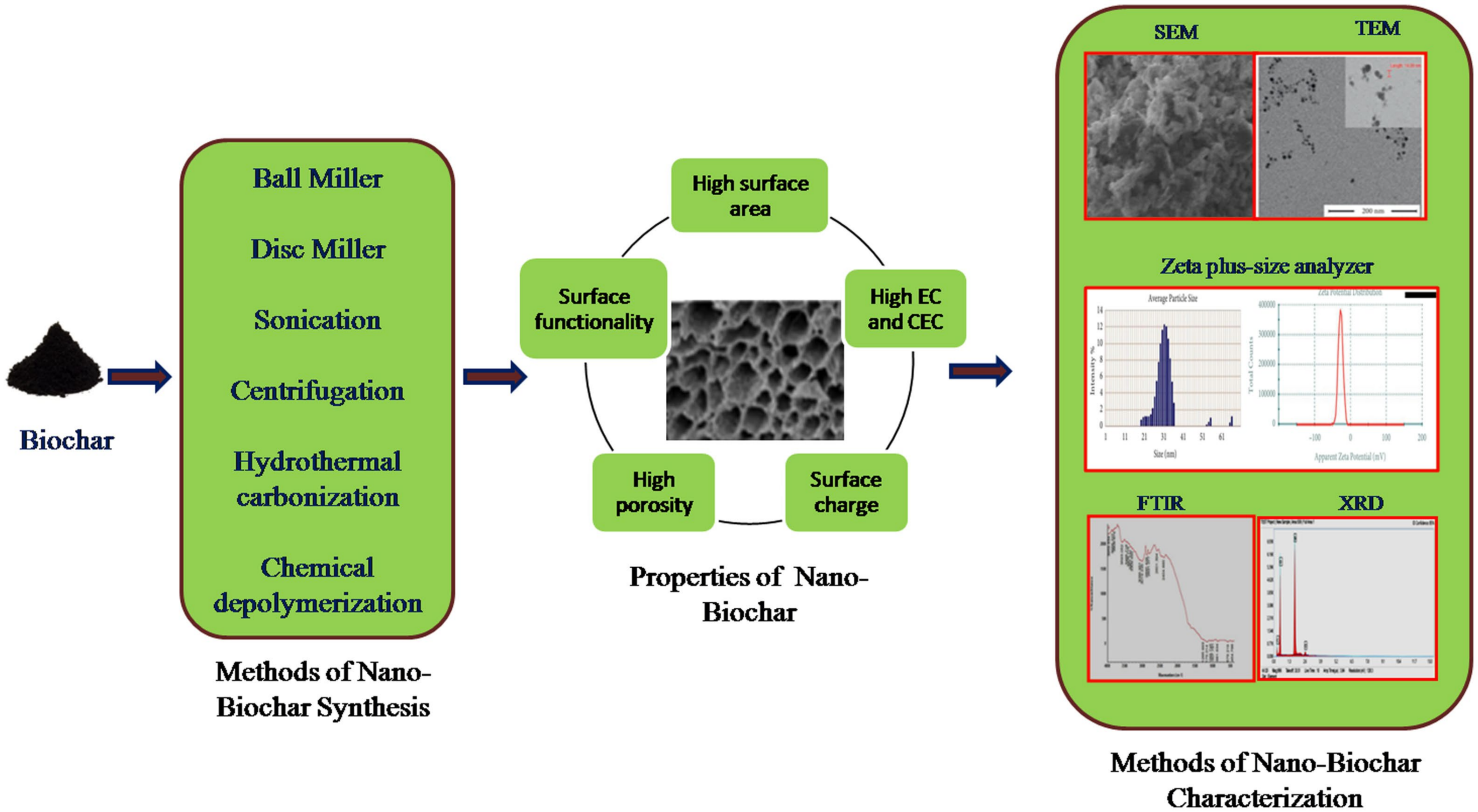
The different approaches for synthesis of nano-BC and its method of characterization.

The intrinsic properties of nano-BC are significant in their selection for wide applications. Plant derived nano-BC have large aromatic cluster size and high oxygen surface functionality resulting in higher affinity and coordinate binding of organic pollutants and heavy metals (Figure 2). Nano-BC fabricated from municipal wastes have abundant carbonate, sulfate and aluminosilicate groups, which enable heavy metal complexation and co-precipitation (Song et al., 2019). Likewise, the degree and type of functional groups and porosity influence nano-BC efficacy as a nano-adsorbent and nano-catalyst. The graphitic and amorphous character of BC (hardness and abrasion resistance) can influence the fabrication, characters and morphological and physiological diversity of nano-BC (Anupama and Khare, 2021). Nano-BC synthesized by bulk-BC fabricated at high-temperature has a higher carbon amount, bulk density, and extractable cations such as Ca, Fe, K, Mg, Mn, P, and Zn (Nath et al., 2019). The carbon amount of nano-BC derived from coconut fibers (90–94%) was greater than that of nano-BC from sewage sludge (4%). Generally, the nano-BC has comparatively greater ash content and lower aromatic and carbonized carbon content than the macro-BC (Wang et al., 2013).

The duration and operating temperature of pyrolysis affect the properties of fabricated nano-BC. An increase in pyrolysis temperature increases nano-BC size owing to improved solid density of micro-BC, resulting in the synthesis of large particle (Zhou et al., 2017). Likewise, increasing pyrolysis duration facilitates the transformation of less dense disordered carbon to small particles that form denser mass fractal architectures (Nath et al., 2019). Nano-BC synthesized at lower temperatures (300–400°C) have smaller surface areas (5.6–47.2 m^2^g^−1^), but nano-BC synthesized at higher temperatures (450–600°C), possess higher surface area (342–430 m^2^g^−1^) due to devolatilization of biomass and generation of surface porosity (Ramanayaka et al., 2020a). The surface area of nano-BC produced by ball milling, sonication, carbonization and centrifugation were in ranges of 3.67–1736, 0.76–264, 9.08–173, and 21.7–253 m^2^g^−1^, respectively (Anupama and Khare, 2021). The zeta potential describes the charge on nano-BC surface and stabilizes the efficiency of nano-BC colloidal solution. The higher zeta potential exhibits lesser particle aggregation and increased dispersion. Nano-BC display greater zeta potential (19.4 to 87 mv) as compared to bulk-BC indicating that nano-BC possesses higher degree of dispersity and colloidal stability.

## 4. Application of nano-BC in environmental remediation

**Figure 3:**
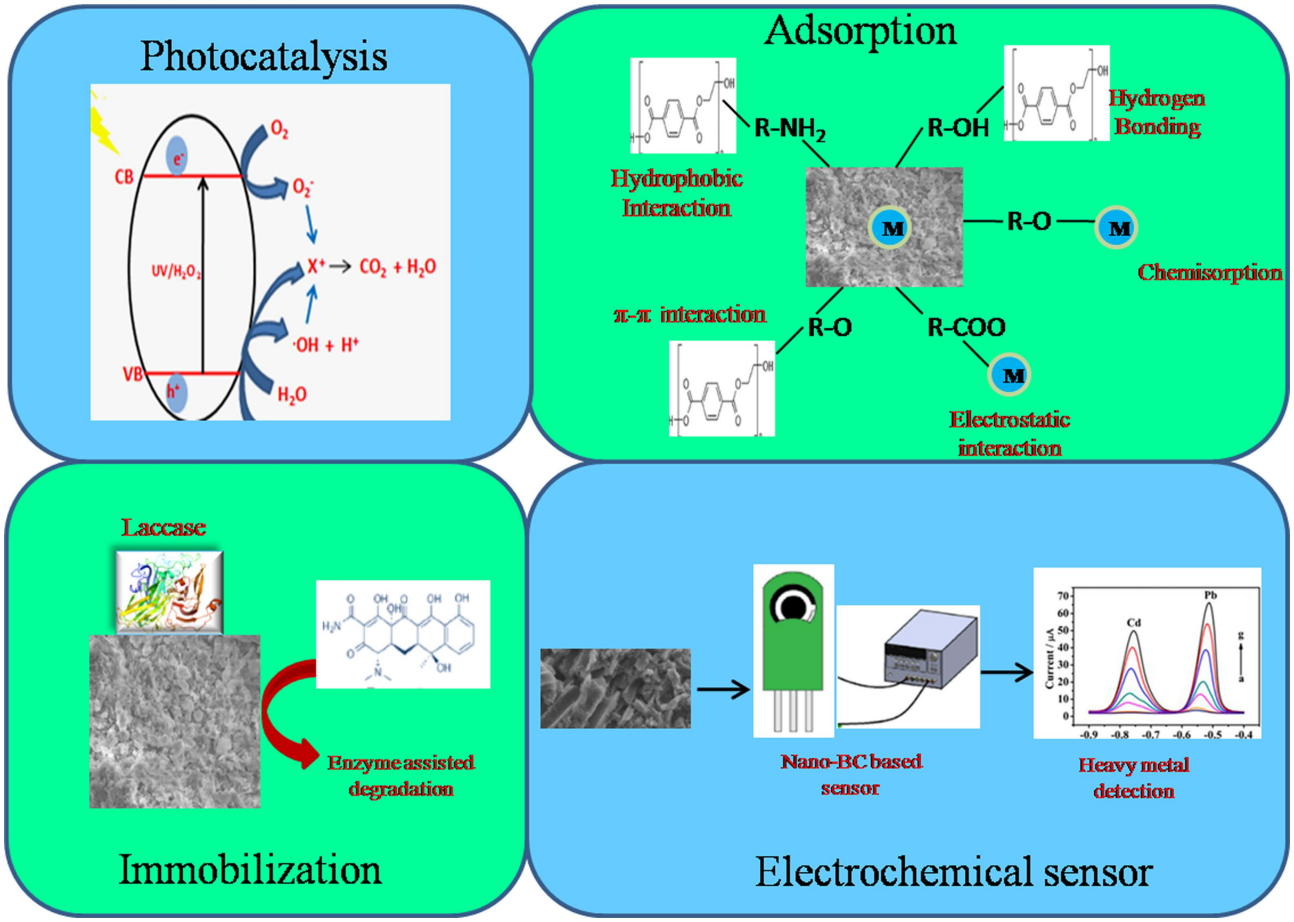
Application of nano-BC in remediation of environment pollutants and their mechanism.

Biochar is recognized as a carbon-negative source since it produces energy while sequestering carbon and has emerged as a potential technology for dealing with several environmental challenges (Jiang et al., 2023). Moreover, the generation of eco-friendly energy and electrodes having enhanced properties using nano-BC is also being explored. Recently, nano-BC is being explored for diverse environmental applications including carbon sequestration, energy generation and treatment of emerging contaminants (agrochemicals, pharmaceuticals, inorganic and organic compounds) from contaminated sites (Figure 3). Nano-BC functions as an excellent adsorbent and thus displays remarkable adsorption capacity for wide range of contaminants (Tables 2, 3). Furthermore, nano-BC also accelerates the breakdown of organic compounds through catalytic electronic shifts like a biocatalyst (Yang et al., 2017).

**Table 2:** Applications of nano-BC for heavy metal adsorption from contaminated environments.

Nano-BC type	Contaminants	Performance	References
Nanosized rice-husk biochar	Fluoride (3–10 mg L^−1^)	90% removal within 60 min	Goswami and Kumar (2018)
Bark chips derived nano-BC	Cu^2+^, Pb^2+^, and Zn^2+^	Sorption rate of 121.5, 336, and 134.6 mg g^−1^ for Cu^2+^, Pb^2+^, and Zn^2+^, respectively	Arabyarmohammadi et al. (2018)
Wood chips derived nano-BC	Cu^2+^	Adsorption rate of 22 mg g^−1^ for Cu^2+^	Safari et al. (2019)
Rice hull derived nano-BC	Cd^2+^	High sorption of Cd^2+^ and reduced uptake and phytotoxicity of Cd	Yue et al. (2019)
Ball milled wheat straw-Biochar	Pb^2+^	Adsorption by ion exchange and precipitation with sorption rate of 134.68 mg g^−1^ for Pb^2+^	Cao et al. (2019)
Rice straw and Palm leave nano-BC	NH^4+^ and H_2_PO_4_	Infinite adsorption capacity for NH^4+^ and H_2_PO_4_	Helal et al. (2019)
Nano-BC	Cd^2+^ (5–300 mgL^−1^) Cr^6+^ (1–25 mgL^−1^)	Higher partition coefficient for the elimination of heavy metals	Ramanayaka et al. (2020a)
Hickory chips Ball-milled nano-BC	Sulfamethoxazole and Sulfapyridine	Removal rates of 83.3% (Sulfamethoxazole) and 89.6% (Sulfapyridine)	Huang et al. (2020)
CuO modified hickory wood chips ball-milled nano-BC	Reactive red dye	Adsorption rate of 1,399 mg g^−1^ for reactive red dye	Wei et al. (2020)
Rice straw nanobiochar (Nano BCs)	Antibiotic resistance genes-amp C, erm B	Adsorption of eDNA-Nano 700 (60%), Nano400 (31.3%)	Lian et al. (2020)
Pine wood nano-BC	Ni	Removal rate of 71% for Ni	Sajjadi et al. (2020)
Sludge derived nano-BC	Pb^2+^ (5 mg L^−1^)	Removal rate of 99.87% for Pb^2+^ at 0.5 g of nano-BC within 30 min	Makshut et al. (2020)
Cornstalk derived nano-BC	Cr^6+^	Elimination rates of 49.6, 65.8, and 97.8% for Cr by Fe^0^-nanobiochar composite consisting of biochar pyrolyzed at 300, 500, and 700°C respectively	Wang et al. (2020)
Ball milled woody nano-BC	Cd^2+^	Adsorption rate of 1062.4 mg kg^−1^ for Cd^2+^	Ramezanzadeh et al. (2021)
Ball milled phosphorus loaded Corn straw nano-BC	Cd^2+^ and Pb^2+^	Adsorption rates of 8.7 mg g^−1^ (Cd^2+^), 126.0 mg g^−1^ (Pb^2+^)	Zhang et al. (2022)

**Table 3:** Applications of nano-BC in remediation of organic pollutant contaminated environments.

Nano-BC type	Contaminants	Mode of action	Performance	References
Ball-Milled sugarcane bagasse nano-BC	Methylene blue (50 mg L^−1^)	Adsorption	Adsorption of methylene blue by π–π interaction and electrostatic attraction with sorption rate of 354 mg g^−1^	Lyu et al. (2018)
Corn straw and rice husk derived nano-BC	Diethyl phthalate	Adsorption	Adsorption rate of 27.65–33.87 mg g^−1^ for diethyl phthalate	Ma et al. (2019)
Rice husk derived nano-BC	Toluene	Adsorption	Adsorption rate of 264 mg g^−1^ for toluene by nano-BC enriched in silicon	Shen and Zhang (2019)
Bamboo	Methylene blue	Adsorption	74% removal of methylene blue by nano-BC	Wang et al. (2019)
Chitosan-nanobiochar composite	–	Nano-biocatalyst	Encapsulated laccase retained 30% of the initial activity after 5 cycles	Naghdi et al. (2019)
Rice husk and wheat straw derived nano-BC	Galaxolide	Adsorption	Adsorption rates of 609–2,098 mg kg^−1^ for galaxolide	Zhang Q. et al. (2019)
Hickory wood	Acetone	Adsorption	Adsorption rate of 103.4 mg g^−1^ for acetone	Xiang et al. (2020)
Wheat straw derived nano-BC	Tetracycline	Adsorption	Tetracycline adsorption rate of nano-BC pyrolyzed at 700°C for was 268.3 mg g^−1^	Li et al. (2020a,b)
Artichoke leaves derived nano-BC	Metformin hydrochloride (10 mg L^−1^)	Adsorption	Removal rates for metformin hydrochloride by modified nano-BC from tap water, wastewater and sea water was 87.0, 97.0, and 92.0%, respectively	Mahmoud et al. (2020)
Poplar woodchips derived nano-BC	Enrofloxacin	Photocatalytic degradation	Degradation rate of 80.2% for enrofloxacin by nano-BC pyrolyzed at 300°C	Xiao et al. (2020)
Date-palm derived nano-BC	Phosphate and nitrate	Adsorption	Highest monolayer sorption rates of 177.97 and 28.06 mg g^−1^ for phosphate and nitrate	Alagha et al. (2020)
ZnO modified nano-BC derived from cellulose nanocrystals	Phenol	Photocatalysis	Photocatalytic removal of 99.8% of phenol within 90 min	Zhang et al. (2021)
Wheat straw and rice husk derived nano-BC	Tetracycline	Adsorption and microbial degradation	Removal rate of 94.9–96% for tetracycline by nano-BC	Sun et al. (2022)
Amberlite cation exchanger (ACE) IR-120 modified *Cynara scolymus* derived nano-BC	Methylene blue	Adsorption	Removal rates of 96.27–99.14% for methylene blue	Mahmoud et al. (2023)
Mulberry waste derived nano-BC	Tetracycline	Adsorption	Removal rate of 103.7% for tetracycline	Yu et al. (2023)

### 4.1. Nano-biochar as an adsorbent

Nano-BC has demonstrated exceptional adsorption capability of hazardous organic and inorganic compounds, personal care products, pharmaceutically active compounds, insecticides, and heavy metals from various environmental matrices (Ma et al., 2022; Jiang et al., 2023; Mahmoud et al., 2023). The excellent sorption ability of nano-BC is due to the generation of a stable colloidal solution, greater surface areas, porosity, and surface charge. Chemical and physical adsorption, precipitation, and ion-exchange are the three primary process described for the adsorption of inorganic pollutants on nano-BC (Amusat et al., 2021). The carboxyl, phenol and hydroxyl groups present on nano-BC surface assist in chemi-sorption of contaminants by exchanging anionic ions with cationic contaminants. Physi-sorption occurs due to electrostatic and Van der Waals interactions among the freely mobile electrons of surface aromatic functional groups of the derived nano-BC, ultimately resulting in non-covalent attraction with C=C bonds (Amusat et al., 2021). Precipitation is also considered as one of the primary processes of sorption of inorganic contaminants. It generally involves heavy metal ion precipitation onto the nano-BC surface either in solid form or in the solvent during the adsorption process. The adsorption of organic contaminants by nano-BC consists of different sorption mechanisms including; electrostatic and hydrophobic interaction, ion exchange and pore–filling (Rajput et al., 2022). Moreover physical sorption is regarded as an initial removal mechanism indicating that nano-BC may transport and subsequently desorb the toxic contaminants from aquatic environment. High temperature pyrolysis enhances the specific surface area and void structure richness of nano-BC and reduces hydrophilic surface functional groups and thus physic-sorption is the prominent mechanism for adsorption. However, the surface area of low-temperature pyrolyzed nano-BC is comparatively low and hydrophilic surface functional groups are high, suggesting chemi-sorption to be the dominant mechanism of adsorption (Jiang et al., 2023).

### 4.2. Nano-biochar as an adsorbent for removal of inorganic compounds

Elbehiry et al. (2022) studied the mono-and multi-sorption of metals (Cd, Cr, and Ni) on water hyacinths and black tea derived BC and nano-BC as a potent, economical and environmentally acceptable absorbents. The nano-BC eliminated >98.8% of Cr and Cd in mono-and competitive systems and the Freundlich isotherm model fitted most appropriately in the sorption kinetics (Elbehiry et al., 2022). The elimination of Cd(II) from an contaminated systems by nano-BC embedded in Ca-alginate beads and fabricated using ball-miller were reported (Wang et al., 2018). The improved surface functionality (oxygen-consisting groups) worked as effective sorption sites, promoting Cd(II)-calcium(II) ion exchange. The pH-dependent variations in Cd(II) sorption revealed the significance of oxygen-consisting surface groups (carboxylic, lactonic, and hydroxyl). The adsorption potential of nano-BC is determined by the surface area, humic acid, functional groups, and graphitic nature. Magnetic nano-BCs (nano zero-valent iron, iron sulfide, and iron oxide BC) display better functionality and magnetic properties that permit nano-BC recovery for recurrent usage. Improved chemical reduction, chemical precipitation, electrostatic interaction, surface complexation, ion exchange and radical activation due to synergistic effect of iron and nano-BC composites, better removal rate for wide range of pollutants are reported (Lyu et al., 2020a; Li et al., 2020b). Sisay et al. (2023) reported that Mg/Zr modified nano-BC derived from spent coffee grounds is an efficient sorbent for phosphate recovery and phosphorous release fertilizer. Furthermore, thiol-modified ball-milled BC demonstrated an improved Hg(II) elimination efficiency, with a sorption rate of 320.1 mg g^−1^, as compared to unmilled BC (105.7 mg g^−1^) (Lyu et al., 2020b). The amino-functional silica-coated magnetic nano-BC derived from *Cynodon dactylon* exhibited improved adsorption rates of 220.4 and 185.4 mg g^−1^ for Cu and Pb, respectively. The nano-composites also demonstrated a 15-fold reuse capability and highest elimination rates for Cu and Pb (Dhanya et al., 2022). The efficacy of ethylenediamine modified nano-BC in the elimination of Cr (VI) and prednisolone was investigated (Mahmoud et al., 2022). Electrostatic, hydrophobic and π-π interaction, ion exchange and complexation are the reported processes for Cr (VI) and prednisolone sorption onto the modified nano-BC. Similarly electrostatic interaction was involved in the elimination of anionic inorganic contaminants from aqueous systems by CuO modified nano-BC. CuO provides cationic sorption sites on surface of nano-BC for the contaminant binding and its subsequent elimination (Wei et al., 2020).

### 4.3. Nano-biochar as an adsorbent for treating organic compounds

Bulk-BC is the most commonly employed sorbent for treating a wide range of toxic contaminants (Yang F. et al., 2020). Nonetheless, nano-BC has an advantage above macro-BC due to larger specific surface area, higher negative Zeta-potential and greater surface functionality (Lian et al., 2018). Ball-milled BC displayed adsorption capabilities of 100.3 and 57.9 mg g^−1^ for removing antibiotics sulfamethoxazole and sulfapyridine, respectively (Huang et al., 2020). According to Shen et al. (2020), any changes in mechanical, physico-chemical, and morphological characters of nano-BC may improve its adsorption efficacy. Luong et al. (2020) described that the change in pH from acid to alkaline using a detergent (tween 80) can increase the sorption capacities of pinewood nano-BC by up to 63%. Xiao et al. (2020) reported that goethite modified peanut shell nano-BC exhibited intercalated hetero-structures and improved hetero-aggregation, which resulted in better adsorption rates. Few reports have employed nano-BC for efficiently removing oxytetracycline from aqueous systems (Li et al., 2020b; Ramanayaka et al., 2020b). The larger surface areas and higher oxygenic groups on nano-BC surface enabled remarkable removal of trichloroethylene with a degradation rate of 99.4% within 5 min, where nZVI-enhanced SO4• synthesis improved the degradation rate (Yan et al., 2015). The removal rate for dimethyl phthalate, diethyl phthalate, and dibutyl phthalate by BC-graphene nanosheets was substantially greater than that of bulk-BC. The aromatic groups on BC graphene nanosheets displayed π–π EDA linkages with the aromatic ring of dimethyl phthalate while hydrophobic groups are involved in dibutyl phthalate binding (Abdul et al., 2017).

The sorption of Cu(II), tylosin and sulfamethoxazole on nano-hydroxyapatite modified BC occurred due to electrostatic and π–π interaction, and hydrogen bonds (Li et al., 2020b). The occurrence of tylosin and/or sulfamethoxazole increased adsorption of Cu(II) significantly (Li et al., 2020b). Nano-BC obtained from hickory wood with specific surface area of 305 m^2^ g^−1^ efficiently adsorbed various compounds (acetone, cyclohexane, chloroform, ethanol, and toluene) with an adsorption rate in the range of 23.4–103.4 mg/g (Xiang et al., 2020). The volatile organic compounds easily diffuse through the pores of nano-BC to reach the interior during sorption, reaching equilibrium after about 1 h. The physico-chemical, morphological and mechanical characters of nano-BC such as pore size, specific surface area, BC composition and contaminant characters are the most important factors in surface sorption (Xiang et al., 2020). The volatile polar organic compounds (acetone, ethanol, and chloroform) are sorbed onto nano-BC by dipole–dipole interaction and hydrogen bonds. The sorption of weakly polar volatile organic compounds exhibited more heterogeneity than the sorption of polar compounds. Furthermore, due to stronger intermolecular forces, organic contaminants with high boiling points (ethanol, cyclohexane, and toluene) were efficiently sorbed by nano-BC. Thus, the sorption of volatile contaminants by ball-milled nano-BC is a highly potent alternative for removal of air pollutants and warranting detailed research into the mechanisms and critical factors (Anupama and Khare, 2021).

### 4.4. Nano-biochar as an immobilization material for enzymes/biocatalysts

Nano-BC can be utilized as an enzyme/microbe/biocatalyst carrier material for achieving continued breakdown of contaminants due to its high mobility and tunable surface chemistry (Table 4). Enzymatic catalysis is a sustainable process for degradation of pollutants and thus laccases immobilized nano-BC is being extensively employed for biodegradation of different contaminants. Oxygen functionalized nano-BC supported Lacasse was utilized for treating carbamazepine contamination (Naghdi et al., 2017). The acidic treatment introduced hydroxyl groups, with its subsequent oxidation to carboxyl groups. The degradation rates of *>*80% for carbamazepine with recyclability for 3 cycles at a comparative removal efficacy were reported (Naghdi et al., 2017). The immobilization process can be improved by addition of cross-linking compounds such as carbodiimide hydrochloride or glutaraldehyde (Naghdi et al., 2018). Naghdi et al. (2019) recently described a hydrogel technique for encapsulating laccase on nano-BC and chitosan. They reported that adding laccase onto nano-BC significantly improved its thermostability (4–70°C) during storage. Lonappan et al. (2018) used laccase immobilized nano-BC to completely remove (100%) diclofenac (2.5 mg L^−1^) in 2 h, which was faster than laccase immobilized carbon nanotube (6 h), and 40% of the efficacy was retained post 5 cycles. More recently, laccase immobilized onto magnetic nano-BC was fabricated and employed for elimination of bisphenol A from aqueous environment (Zhang et al., 2020). The complete elimination of bisphenol A (25 mg L^−1^) was reported within 75 min and 85% efficacy of the composite remained after 7 cycles. Cold-active toluene*/o*-xylene monooxygenase and catechol 1,2-dioxygenase were immobilized onto micro/nano-BC or chitosan and employed for petroleum hydrocarbon degradation (Miri et al., 2021). The results suggested that immobilization improved the storage stability of the enzymes (>50% recyclability after 1 month at 4°C) and degradative ability (>80% degradation of BTEX).

**Table 4:** Recent studies on application of nano-biochar and biochar based nano-composites.

Nano-composite	Application	Outcomes	References
Ball-milled wheat straw nano-BC	Adsorbent for removal of Cd^2+^	Nano-BC acted as double-edged sword for Cd^2+^ adsorption on zeolite	Cao et al. (2023)
Ball-milled bone nano-BC	Adsorbent for removal of Cu^2+^, Pb^2+^, Cd^2+^ Mn^2+^	Reduction in heavy metal bioavailability and improved N and P soil fertility	Xiao et al. (2023)
Ball-milled corn stalk nano-BC	Adsorbent for removal of Cd^2+^	Adsorption by Precipitation and complexation; threefold enhanced adsorption of cadmium in comparison to bulk BC	Ma et al. (2022)
Metal oxides coated biochar nanocomposites	Adsorbent for removal of textile dyes in constructed wetlands	Significant removal of Reactive Golden Yellow Merl dye	Munir et al. (2023)
CoFe_2_O_4_-BC Nano-composite	Adsorbent for removal of Methylparaben	Adsorption rate of 85.6% for methylparaben; Vander Waals forces, H-bonding, and dipole interaction involved in adsorption	Fito and Nkambule (2023)
Zero-valent iron supported BC	Adsorbent for removal of Organochlorine pesticide	Removal efficiency up to 92%	Batool et al. (2022)
Ag/biochar nano-composites	Catalytic degradation of p-nitrophenol	98% degradation of p-nitrophenol to p-aminophenol	Behera et al. (2022)
Sugarcane pressmud nano-BC	Plant growth promotion in Cr contaminated soil	Reduced Cr toxicity and improved plant (black cumin) growth	Ramzan et al. (2023)
Ball milled BC	Electrochemical sensor for monitoring of Pb^2+^ and Cd^2+^	Detection limit: 5.86 Fm, 0.883 aM for Pb^2+^ and Cd^2+^ respectively	Mao et al. (2022)
Ag–Cu/biochar	Removal of doxycycline	Removal rate of 81% after 6 repeated cycles	Hosny et al. (2022)
Copper/egg shell BC nano-composite	Electrochemical sensor for detection of nitrite	Broader linear range, detection limit:0.63 μM, and high sensitivity	Cao et al. (2020)
Nano-BC particle	Electrochemical immunosensor for detection of microcystin-LR toxin in water	Response time ≈ 5 min, detection limit: 17 pM	Yao et al. (2021)
ZnO BC nano-composite	Electrochemical sensor for detection of bisphenol A	Detection limit: 1 × 10^−7^ mol/L, detection sensitivity: 92 mA/M.	Hu et al. (2022)
BC nano-composites	Electrochemical sensor for detection of 17β-estradiol	Detection limit:11.30 nM	Dong et al. (2018)
Nano-BC and Bioinoculant	Mitigation of antibiotic resistance genes (ARG) in Cu contaminated soil	Impeded transport of ARGs in plant, reduced bioavailability of Cu, Improved plant growth	Duan et al. (2023)
Corn cob nano-BC	Laccase immobilization	Immobilization rate: 99.60% and activity: 22.54 U mg^− 1^	Borges et al. (2023)

### 4.5. Nanobiochar as photocatalyst

In recent times nano-BC supported photocatalysts have been fabricated through different methods to photo-catalytically breakdown water pollutants (phenolics, dyes, pharmaceutically active components, antibiotics). The photocatalysis is dependent on the methods of nano-BC synthesis. BC is an excellent support for photocatalysts owing to its tunable functional groups, chemo-stability, and electrical conductance. BC as a photocatalytic support reduces e−/h + recombination and thus displays increased catalytic efficiency (Ahmaruzzaman, 2021). Recently, the nano-BC/ZnO photocatalyst derived from carbon/ZnO nanocomposite were employed for photocatalytic breakdown of phenol (Zhang et al., 2020). *In-situ* precipitation and carbonization were used to create the photocatalyst, with carbon nano-composites serving as both the template and the carbon source. These composites demonstrated remarkable stability and durability with photodegradation rate of 95% suggesting that the integration of carbon nano-composites could efficiently reduce the photo-corrosion of ZnO (Zhang et al., 2020). The BC caused a reduction in the band gap of ZnO through continuous electron/hole separation and transport, increasing phenol degradation rates. A unique core-shell P-laden BC/ZnO/g-C3N4 nano-composite was recently reported as an excellent catalyst for atrazine breakdown and a potential regulated-release nanofertilizer for enhancing P utilization rates (An et al., 2022). The results revealed the formation of a Z-shaped heterojunction between ZnO and g-C_3_N_4_ in Pbi-ZnO-g-C_3_N_4_. The authors suggested that BC functions as an electron-transfer agent promoting the disjunction of electron–hole pairs. Highest atrazine photodegradation rates of 85.3% within 260 min were reported (An et al., 2022).

### 4.6. Nano-biochar used in electrochemical biosensor

The electrochemical characteristics of nano-BC have recently received attention for its potential application as an alternative to carbon electrodes. The high adsorption capacity of nano-BC enables selective entrapment of chemicals to improve their concentration on electrode surface thus increasing the sensitiveness of electrochemical biosensors for detection (Table 4). The water-dispersible nature of nano-BC allows its use in film-forming methods for creating film electrodes with potent electrochemical applications (Plácido et al., 2019). In supercapacitors also, the electrode materials have recently been substituted with nano-BC due to its both meso and microporous structures having higher specific surface area resulting in improved performance. Biochar is also being used to replace cathode materials in batteries. Water dispersible nano-BC has been successfully used as an electrode material for Pb(II) and Cd(II) voltammetric sensors (Liu et al., 2016; Li et al., 2017). Li et al. (2017) investigated the impact of loading a hybrid of both bulk and nano-BC on the electrodes of a voltammetric sensor for detection of Pb^2+^, and increased sensitivity and electric current (3.24–4.0 and 4.5 μA) were reported. Moreover, fluorescence assay confirmed that BC releases dissolved organic matter containing fluorescent humic compounds (Hernandez-Soriano et al., 2016). The fluorescent characters of nano-BC have also been exploited for development of fluorescent detectors for metals (Plácido et al., 2019). Nano-BC fabricated from sorghum and rice straw and dairy manure was utilized as a probe for heavy metal detection and the results revealed a 100, 66, 66, and 33% accuracy for Pb^2+^, Ni^2+^, Cu^2+^, and Hg^2+^ detection, respectively, (Plácido et al., 2019). This was the initial report to suggest the application of nano-BC quenching data as a simple and accurate method for detection of toxic metal ions.

Furthermore, functionalized magnetic baggase nano-BC was fabricated by combining carboxyl groups and enzymes for bisphenol A monitoring in aqueous systems, demonstrating high sensitiveness as well as excellent electrochemical activity. Dong et al. (2018) studied the possibility of loading nano-BC on glass carbon electrodes for 17-estradiol monitoring and the electric current increased from 0 to 1.5 μA at the 17-estradiol amount of 3 M. The transfer resistance was lowered from 495 to 325 Ω on loading electrodes with nano-BC fabricated at 800°C in comparison with pristine BC resulting in increased electrode conductance. Ball-milled BC modified carbon electrodes displayed remarkable electrochemical characters and electrocatalysis as indicated by conductance, peak-to-peak disjunction, resistivity, and charge transfer resistance (Lyu et al., 2019). He et al. (2020) employed tyrosinase immobilized magnetic nano-BC for detection of bisphenol A. The developed electrochemical biosensor demonstrated a monitoring range of 2.78 nM with a linear range of 0.01–1.01 M, and the sensitivity remained consistent after 8 cycles without signal reduction. The use of nano-BC in electrochemistry can be expanded into new fields such as biomass electrocatalysis, fuel cells, and CO_2_ reduction. A novel electrochemical biosensor for detection of lead and cadmium was synthesized by fabricating a high conductance and contaminant specific electrode. Ball milled BC was employed as the conductive material having large conductance, oxygen rich functionality and pores of ion-imprinted polymer functioned as target interacting sites (Mao et al., 2022). The ion imprinted bulk-BC electrode was created by *in situ* electro-polymerization of L-Cysteine and template metal ions on glassy carbon-modified bulk-BC, followed by template removal. The electrode detected very low concentrations of lead and cadmium using anodic dissolved differential pulse voltammetry. The monitoring range of 5.86 fM and 0.883 aM, and linear ranges of 25 fM ~ 1 μM and 0.1 fM ~ 1 μM, respectively were reported. The electrodes displayed no interaction with other ionic and organic molecules, and could be recycled for 7 cycles without losing detection sensitivity (Mao et al., 2022). Ferlazzo et al. (2023) described an electrochemical biosensor fabricated by nano-BC for the detecting nitrites and sulfites in contaminated water bodies. The nano-BC was placed on a commercial screen-printed carbon electrode (SPCE). The fabricated sensor outperformed the normal SPCE sensor in terms of detection limits and electrochemical oxidization of sulfites and nitrites in water (Ferlazzo et al., 2023).

## 5. Factors affecting performance of nano-BC for environmental remediation

The biogeochemical nature of nano-BC during contaminant elimination is influenced by a variety of physico-chemical parameters (Table 5). Nano-BC possessing high cationic surface groups allows improved ion exchange capacity of nano-BC with toxic metal ions. A higher concentration of aromatic groups on surface of nano-BC results in better π-π-interactions with organic contaminants (Jiang et al., 2023). The aggregation capability, suspension stability, and electrokinetic characters of nano-BC impact the sorption of contaminants that may be determined by the zeta potential (Filipinas et al., 2021). The surface functionality is also dependent on pyrolysis temperature used for BC synthesis and low pyrolytic temperature was found to have abundant surface functional groups, higher zeta potential and stronger colloidal stability (Xu et al., 2020). In case of metal ions, the valency, hydration area, electronegative nature and hydrolytic constant are the dominant parameters that influence the metal ion removal by nano-BC. Zhang et al. (2022) reported that the sorption rate of nano-BC for Pb^2+^ was significantly higher than that for Cd^2+^ in same treatment conditions. The authors suggested that variation in metal characteristics (hydration area, hydrolytic constant) and their affinity for binding sites are responsible for different sorption rates. For organic contaminants, groups such as polar, hydrophobic, aromatic, and molecular weight of the contaminant affect their interaction with nano-BC. In general, the sorption of highly hydrophobic compounds by carbonaceous compounds is slow (Choi et al., 2014). Galaxolide is a highly hydrophobic contaminant and therefore shows high sorption to ball milled nano-BC (Zhang Q. et al., 2019). Moreover, high molecular weight contaminants are hardly sorbed by nano-BC due to size exclusion and pore filling effect resulting in restriction of these compounds from entering small pores on nano-BC (Zhu et al., 2022). Nano-BC assisted remediation of contaminants is also influenced by environmental parameters including: pH, soil microbes, dissolved organic matter, root exudates and coexisting contaminants (Jiang et al., 2023). In lower pH environments, the surface functional groups of nano-BC get protonated to generate H^+^. This causes competition between H^+^ and cationic contaminants for sorption sites thus affecting nano-BC’s sorption capability (Mahmoud et al., 2023). Wang et al. (2017) reported that co-existence of lead and p-nitrophenol improved the sorption of p-nitrophenol on nano-BC. In soil systems, carbon of nano-BC may functions as a source of nutrition to soil microbes, thus improving their metabolism and contaminant degradation efficacy (Mukherjee et al., 2022). The root exudates released from plant in contaminated soils can also impact the physical and chemical characteristics of nano-BC thus influencing its contaminant sorption ability (Li et al., 2019).

**Table 5:** Factors affecting the performance of nano-biochar for environmental applications.

Parameter	Impact on environmental application of nano-BC	References
Nano-BC synthesis method		
Ball milling	Fabricated Nano-BC has high specific surface area, pore volume	Anupama and Khare (2021)
Sonication	Better purity and uniform shape of nano-BC	
Centrifugation	High stability nano-BC colloids	
Chemical methods	Higher surface functionality of nano-BC	
Nano-BC properties		
Pyrolysis temperature	High pyrolysis temperature increases specific surface area of BC thus increasing adsorptive capacity	Liu et al. (2018) and Lyu et al. (2018)
	Increase in pyrolysis temperature decreases surface functionality and thus reduces adsorptive capacity	Xu et al. (2020)
	Increase in pyrolysis temperature increases the ash and carbon content of nano-BC	Jiang et al. (2023)
Zeta potential	Zeta potential of nano-BC < micro/macro-BC	Song et al. (2019)
Raw material	Hemicellulosic biomass> lignin results in higher specific surface area of nano-BC	Jiang et al. (2023)
Surface functionality	High cationic groups increase ion exchange capability with metal ions; more aromatic groups allow π–π interactions among nano-BC and organic contaminants	Jiang et al. (2023)
Pollutant characteristics		
Heavy metal	High hydration radius and hydrolysis constant of metal ion reduce their adsorption onto nano-BC; high electronegativity of metal ion result in better binding capacity onto nano-BC	Ni et al. (2019) and Zhang et al. (2022)
Organic contaminant	Highly polar organic contaminant readily adsorb onto nano-BC; more hydrophobic group lowers adsorption; large molecules are less readily adsorbed by nano-BC	Zhuang et al. (2021) and Zhu et al. (2022)
Environmental factors		
pH	Acidic pH reduces adsorptive capacity of nano-BC	Mahmoud et al. (2020)
Extracellular secretions by microbes	Extracellular polymers of microbes block the pores of nano-BC and thus reduce adsorption rate	Zhang et al. (2020)
Root exudates	Oxalic acid increase adsorption of phenanthrene by BC	Li et al. (2019)

## 6. Challenges and environmental concern of N-BC

Nano-BC and colloidal BC possess high surface area, pore size, and surface functionality thus demonstrating remarkable contaminant removal efficiency as compared to pristine BC (Ramanayaka et al., 2020b). However there are several constraints to using native nano-BC in environmental applications including low yield and stability, easy mobilization, high agglomeration, uptake, accumulation, toxic nature and limitations in recovery (Liu et al., 2018). Functionalizing nano-BC with appropriate redox functional groups promotes its stability/suitability for contaminant removal in different environmental matrices but these studies are still in infancy and broad understanding is still necessary for on-site application. The inter-linkages among BC structure, oxygenic surface functional groups, feedstock types and pyrolytic parameters must be assessed for postulating molecular mechanisms of contaminant removal by electrochemical reaction pathways (Amusat et al., 2021). The technologies for large-scale fabrication of nano-BC need to be developed for achieving high yield of nano-BC for wide applications. The green and biogenic methods for fabricating nano-BC need to be investigated for reducing the risk of cross-contamination of chemicals used for synthesis during wastewater treatment. Nano-BC showed improved results when compared to the bulk-BC, however, the comparative performance to other nanomaterials and carbon based nanocompounds should be investigated further (Rajput et al., 2022). The economical assessment of the expenses is critical in evaluating the manufacture and deployment of the nano-BC for wide application. The lack of data on large level synthesis and employment of nano-BC makes estimating the economic aspect of nano-BC difficult. Furthermore, the utilization of wide range of raw feedstock material and different factors and processes for the fabrication of bulk-BC and the lack of standard procedures limit the cost analysis of nano-BC synthesis. The higher adsorption of contaminants and transportability of nano-BC, can cause the risk of cross-contamination of different ecosystems. High dispersion of nano–BC in natural aquatic systems can further expose different organisms to nanoparticle-associated risks (Freixa et al., 2018). The examination of toxic impact on respiratory system indicated a lower risk to human health (Dong et al., 2019). A very limited number of reports suggested the toxic impact of nano-BC and nanocarbon compounds on plant, mammals and soil microflora, thus in spite of their numerous benefits, the eco-toxicity must be investigated (Zhang K. et al., 2019; Rajput et al., 2022).

## 7. Conclusion

Nano-BC is an emerging and potential alternative to carbon-based nanomaterials for wide applicability in comparison to the pristine and bulk-BC. Nano-BC exhibits exceptional physicochemical characters such as high surface functionality and ease of surface modification. It is generally manufactured using ball miller, ultra-sonication, carbonization, centrifugation, and manual grinders. Ball milling is an economical, sustainable and green method, while ultrasonication is more energy-intensive and non-eco-friendly. Nonetheless, process optimization, eco-toxicity, and life cycle evaluation of nano-BC fabrication is necessary for individual process prior to its selection for commercial-scale synthesis. The distinctly minute size of nano-BC offers improved surface areas imparting it with the potential of applicability in environmental remediation. Nano-BC efficiently reduced toxic organic and inorganic pollutants from different environmental matrices as compared to bulk-BC. Nano-BC functions as detoxicant, playing vital part in waste management, reduction of soil erosion, and preventing nutrient loss from soil. The surface features of nano-BC allow it to function as carrier for the immobilizing enzymes, biocatalysts and microbes. Nano-BC is also a potential alternative to chemical electrode and thus functions as biosensor for detection and monitoring of toxic contaminants. Additionally the high surface area also provides habitat for microorganisms on nano-BC, thus understanding their interactions at molecular and genetic level can unfold new areas for hybrid remediation strategies, which however warrants future research. However, there is a considerable knowledge gap about the parameters of the nano-BC synthesis by different methods and their physicochemical characters. The optimization of process parameter for desirable properties (porosity, surface area and functionality and binding sites) and yield is required. Methods for rapid valorization of nano-BC and their transport and distribution into different ecosystems need to be studied for restricting the detrimental impact.
